# Lipopolysaccharide directly inhibits bicarbonate absorption by the renal outer medullary collecting duct

**DOI:** 10.1038/s41598-020-77363-w

**Published:** 2020-11-25

**Authors:** Shuichi Tsuruoka, Jeffrey M. Purkerson, George J. Schwartz

**Affiliations:** 1 Department of Medicine, Tajirigaoka Hospital, Hitachi, Ibaraki Japan; 2 University of Rochester Medical Center, 601 Elmwood Avenue Box777, Rochester, NY 14642 USA

**Keywords:** Physiology, Nephrology, Urology

## Abstract

Acidosis is associated with *E. coli* induced pyelonephritis but whether bacterial cell wall constituents inhibit HCO_3_ transport in the outer medullary collecting duct from the inner stripe (OMCDi) is not known. We examined the effect of lipopolysaccharide (LPS), on HCO_3_ absorption in isolated perfused rabbit OMCDi. LPS caused a ~ 40% decrease in HCO_3_ absorption, providing a mechanism for *E. coli* pyelonephritis-induced acidosis. Monophosphoryl lipid A (MPLA), a detoxified TLR4 agonist, and Wortmannin, a phosphoinositide 3-kinase inhibitor, prevented the LPS-mediated decrease, demonstrating the role of TLR4-PI3-kinase signaling and providing proof-of-concept for therapeutic interventions aimed at ameliorating OMCDi dysfunction and pyelonephritis-induced acidosis.

## Introduction

Acute pyelonephritis is a common, serious bacterial infection of the kidney. The infection often ascends from the bladder and reaches the kidney via the medullary collecting duct. The OMCDi, which is one of the first nephron segments encountered by ascending bacteria, is comprised of principal cells and alpha-intercalated cells (α-ICs)^1^. Urine acidification (bicarbonate reabsorption) by α-ICs is mediated primarily by an apical B1-V-ATPase and a basolateral chloride/bicarbonate exchanger (SLC4A1, AE1)^2,3^.

Tissue acidosis is associated with bacterial infections and the ensuing inflammation^4^. Metabolic acidosis is frequently observed in critically ill patients with sepsis^5^. Sepsis-associated acidosis may be explained, at least in part, by signaling induced by Pathogen Associated Molecular Patterns (PAMPs) via pattern recognition receptors (PRRs) expressed on renal tubule epithelial cells^6^. Good and Watts^7^ have reported that lipopolysaccharide (LPS), the dominant bacterial cell wall constituent of Gram-negative bacteria inhibits bicarbonate reabsorption in the medullary thick ascending limb, and that this effect was likely mediated via TLR4 and phosphoinositide 3-kinase (PI3-K) and their downstream effectors^8^. The effect of LPS was absent in thick limbs from TLR4^−/−^ mice, confirming that the response was mediated by signaling via the TLR4 receptor^7^.

*E. coli* induced pyelonephritis is also frequently associated with metabolic acidosis^9–11^. Whether bacterial derived PAMPs directly affect tubular transport in the OMCDi has not been established. The potential impact of *E. coli* pyelonephritis on urine acidification was examined by studying the effect of LPS and monophosphoryl lipid A (MPLA) on HCO_3_ absorption in isolated perfused rabbit OMCDi. MPLA is a chemically modified derivative of LPS that retains the beneficial immunomodulatory properties of LPS without any inherent toxicity^12^. Such protection against LPS toxicity, can be exploited in studies of LPS effects on renal tubular function^12^. Indeed, MPLA pretreatment eliminated LPS-induced inhibition of HCO_3_ absorption in isolated perfused rat medullary thick ascending limbs^12,13^. Results presented herein demonstrate that LPS exposure inhibits bicarbonate absorption in the OMCDi, and that this effect can be blocked by basolateral (blood-side) administration of MPLA as well as by Wortmannin, a PI3-K inhibitor.

## Methods

### Animals

Female New Zealand white rabbits weighing 1.8–3.2 kg were maintained on standard laboratory chow (Japan Clea) with free access to water^14^ in accordance with protocols and regulations approved by the IACUCs of the Institutional Animal Care Facility of Jichi Medical University and the University of Rochester School of Medicine. Animals were euthanized by intracardiac injection of 130 mg pentobarbital sodium after premedication with ketamine (44 mg/kg) and xylazine (5 mg/kg). Urine was obtained postmortem by bladder tap; urine pH was alkaline, compatible with a standard rabbit diet^15^.

### Microperfusion

Collecting ducts were microdissected at 10 °C from the outer stripe of the outer medulla of the rabbit kidney as described previously^16^. The average perfused length of tubule was 0.65 (SD 0.09) mm. Equilibration and transport were performed using Burg’s solution in the perfusate and bath, containing (mM): 120 NaCl, 25 NaHCO_3_, 2.5 K_2_HPO_4_, 2 CaCl_2_, 1.2 MgSO_4_, 5.5 d-glucose, 1 trisodium citrate, 4 Na lactate, and 6 l-alanine, 290 mOsmol/kg water and gassed with 94% O_2_/6%CO_2_, yielding a pH of 7.4 at 37 °C^15–17^. Bath was continually exchanged at 14 ml/h by a peristaltic pump. Luminal perfusion rate was maintained at 2–2.5 nl/min.

### Bicarbonate transport and transepithelial voltage

Triplicate collections of 15–20 nl of tubular fluid were made under water saturated mineral oil and analyzed for [HCO_3_] using a Nanoflo microfluorometer (World Precision Instruments, Sarasota, FL)^15–17^. Net [HCO_3_] transport was calculated as J_HCO3_ = (C_o_–C_L_) × V_L_/L), where C_o_ and C_L_ are the HCO_3_ concentrations of perfused and collected fluid, respectively, V_L_ is the rate of collected fluid, and L is the length of the tubule (mm), and no water is net absorbed. When J_HCO3_ > zero, there is net HCO_3_ absorption. Transepithelial voltage (mV) was measured using the luminal perfusion pipette as an electrode. The voltage difference between calomel cells connected via 3 M KCl agar bridges to perfusate and bath was measured with a high impedance electrometer.

### Reagents

Lipopolysaccharide (LPS) from E. coli 0111:B4 was obtained from Merck KGaA (Germany, L3024), prepared as a stock solution in DMSO at 50 µg/ml, and diluted into luminal or bath Burg’s solution to a final concentration of 500 ng/ml^12,13^. OMCD were pretreated with LPS for 30–45 min prior to tubular fluid collections. Monophosphoryl lipid A (MPLA) was obtained from Merck KGaA (Germany, 699800P), prepared in a stock solution in DMSO of 5 mg/ml, and diluted into Burg’s solution to 100 µg/ml. The final concentration of MPLA in the bathing solutions was 1 μg/ml^12^. After removal of luminal LPS we allowed 45 min for re-equilibration before resuming collections. Wortmannin was purchased from Merck (Sigma-Aldrich (681675) and dissolved in DMSO to make a stock of 500 μM.

## Results

Rabbits weighed on average (± SE) 2.45 ± 0.08 kg and the urine pH was 8.0 ± 0.02 units. The length of perfused tubules averaged 0.65 ± 0.02 mm. Tubules were perfused at 2–3 nl/min.

### Basolateral LPS inhibits HCO_3_ absorption in OMCDi

**Figure 1:**
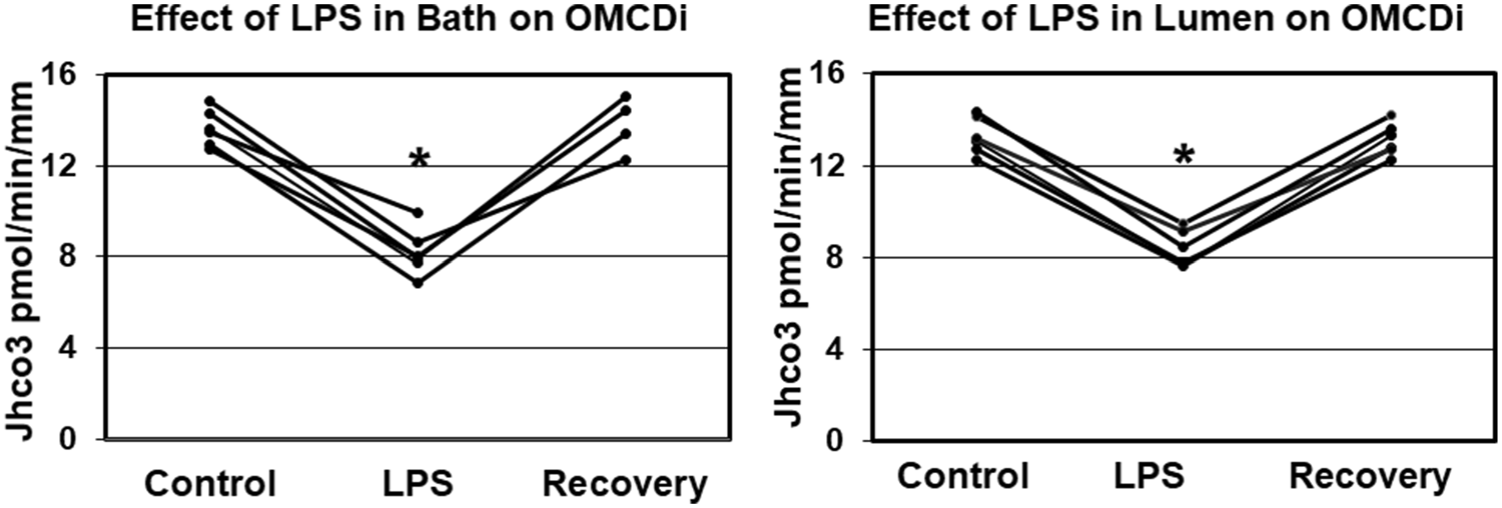
LPS added to the bath (L panel) or to the lumen (R panel) at 500 ng/ml inhibits HCO_3_ absorption by ~ 40% in OMCDs from the inner stripe (n = 6 for each panel).

Basolateral LPS exposure is a model for investigating the impact of sepsis on renal tubular transport mechanisms^6^. Baseline HCO_3_ transport by the OMCDi averaged 13.65 ± 0.33 pmol/min per mm length and the transepithelial voltage was + 3.7 ± 0.2 mV. LPS applied to the bath (500 ng/ml) resulted in a 40% decrease in HCO_3_ transport to 8.22 ± 0.42 pmol/min per mm (p < 0.001) (Fig. 1, L panel), and there was a corresponding fall in transepithelial voltage to 3.1 ± 0.2 mV (p < 0.001). Removal of LPS resulted in a complete return of transport to 13.79 ± 0.61 pmol/min per mm and voltage (+ 3.6 ± 0.2 mV), neither significantly different from control values.

### Luminal LPS inhibits HCO_3_ absorption in OMCDi

Luminal exposure to LPS models *E. coli*-induced pyelonephritis’ impact on HCO_3_ transport by OMCDi. Baseline HCO_3_ absorption was decreased by 37% by luminal LPS at 500 ng/ml (absorption was 13.28 ± 0.32 pmol/min per mm and decreased to 8.37 ± 0.32, p < 0.001) (Fig. 1, R panel). Transepithelial voltage was + 2.7 ± 0.2 mV and decreased to + 2.2 ± 0.2, p < 0.001 (Table 1). The inhibition by LPS was reversible by removing it from the luminal fluid. Recovery HCO_3_ absorption was 13.14 ± 0.29 pmol/min per mm, 99% of control rate and voltage returned to + 2.6 ± 0.2 mV. The recovery transport rate was not significantly different from control (p > 0.5), and the voltage recovered to 97% of control, slightly but significantly less than control (p = 0.02).

**Table 1:** Effect of luminal LPS on transepithelial voltage in the presence of specific agents in the bathing solution.

Bath agent	Control	LPS	Recovery	SE
None	2.7	2.2^a^	2.6	0.2
MPLA	2.2	2.2	2.2	0.1
Wortmannin	2.6	2.4^a^	2.6	0.1

Data given as mean with approximate SE in R column.

^a^Significantly different from mean of control and recovery periods, p < 0.01.

### Basolateral MPLA blocks the luminal LPS effect

**Figure 2:**
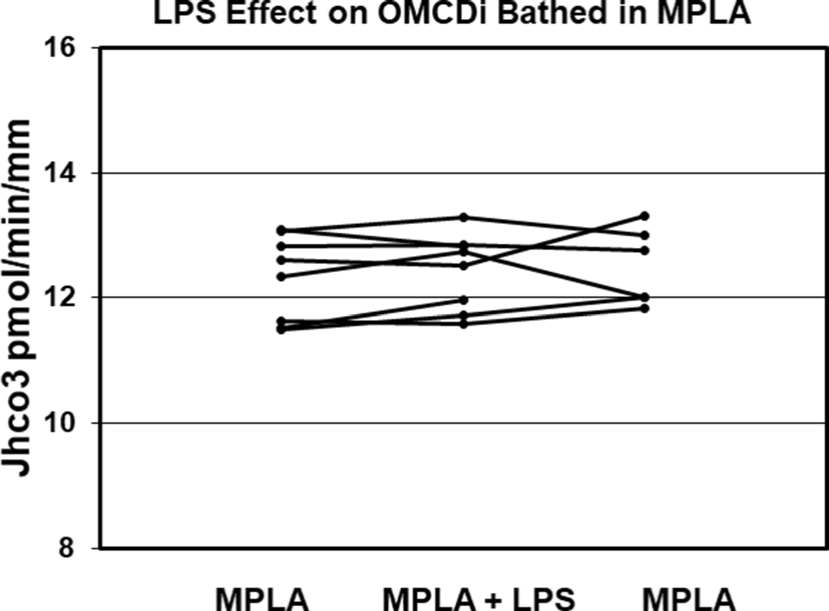
LPS added to the lumen at 500 ng/ml has no inhibitory effect on HCO_3_ absorption by OMCDs from the inner stripe in the presence of 1 μg/ml MPLA in the bath (n = 6 OMCDs).

To confirm and extend results of Watts et al.^12,13^ the effect of MPLA on bicarbonate transport and the LPS response was examined. In two independent experiments, MPLA at 1 μg/ml in the bath had no major effect on HCO_3_ transport (11.74–11.52 and 12.79–13.09 pmol/min per mm) or transepithelial voltage (2.3–2.2 and 2.1–2.0 mV). In the next 6 experiments, MPLA was added to the bath as control, and then LPS to the lumen at 500 ng/ml as the experimental period. LPS was then removed and recovery values obtained. In 6 experiments the HCO_3_ transport rate was not significantly changed during MPLA control, LPS added to lumen, or MPLA post control (12.3 ± 0.3 pmol/min per mm to 12.4 ± 0.3 to 12.5 ± 0.3, respectively, p NS) (Fig. 2); nor was there any significant change in transepithelial voltage (2.2 ± 0.1 mV to 2.2 ± 0.1 to 2.2 ± 0.1, respectively, p NS, Table 1). These results demonstrate that LPS induced luminal signaling inhibits bicarbonate reabsorption in OMCDi and that this effect can be blocked by MPLA on the basolateral surface.

### Basolateral wortmannin blocks the luminal LPS effect

**Figure 3:**
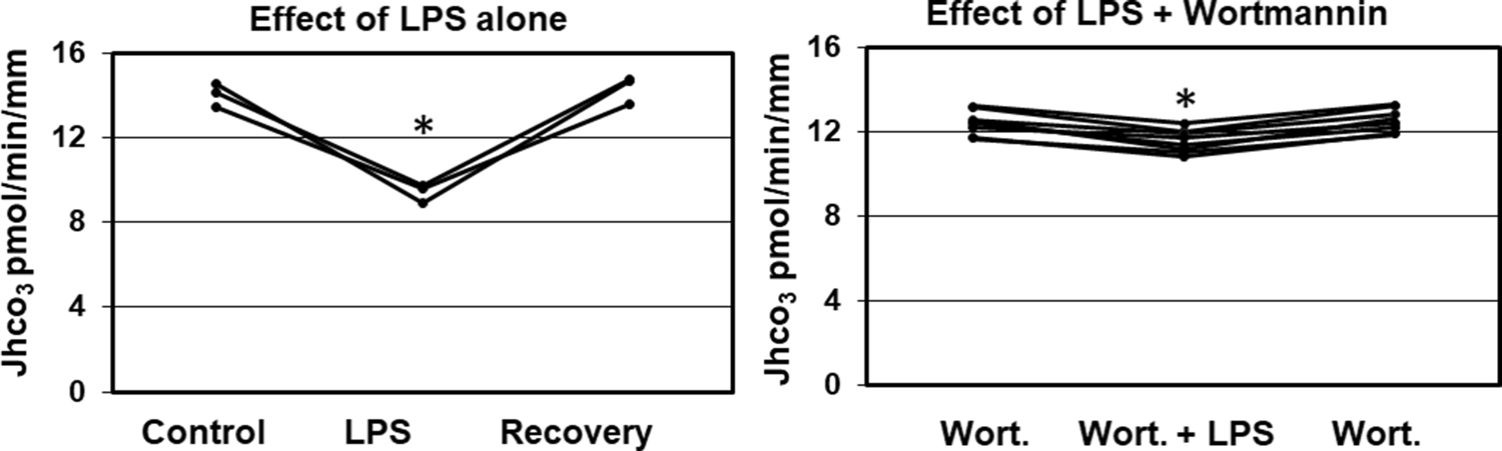
LPS added to the lumen at 500 ng/ml has less inhibitory effect on HCO_3_ absorption by OMCDs from the inner stripe in the presence of 100–200 nM Wortmannin in the bath (R panel, n = 8 OMCDs) compared to LPS alone (L panel, n = 3 OMCDs).

In the mTAL luminal exposure to LPS inhibits basolateral sodium hydrogen exchange via a phosphoinositide 3-kinase (PI3-K)-dependent pathway^8^. In the next eight experiments we examined the effect of PI3-K inhibitor Wortmannin^18^ on LPS-mediated inhibition of bicarbonate absorption in the OMCDi. Similar to the design above for the MPLA studies, wortmannin (100–200 nM) was added to bath during the control period, and then LPS added to lumen (500 ng/ml) for an experimental period, after which LPS was removed and recovery bicarbonate flux was measured in the presence of wortmannin. In eight independent experiments (Fig. 3, R panel) pretreatment with Wortmannin blocked the effect of LPS; HCO_3_ transport in OMCDi treated with LPS + wortmannin was 93 ± 1% of control and recovery values (this change was still significantly different from zero, mean of control and recovery was 12.5 ± 0.2 pmol/min per mm compared to LPS of 11.6 ± 0.2 pmol/min per mm, p < 0.001). In addition, there was only a small but significant decrease in transepithelial voltage such that the voltage during LPS + Wortmannin was 95 ± 1% of control and recovery values (mean of control and recovery was 2.6 mV compared to LPS voltage of 2.4 mV, p < 0.001, Table 1). In the absence of wortmannin, luminal LPS inhibited the HCO_3_ absorptive flux by 34 ± 3% (Fig. 3, L panel, n = 3, mean of control and recovery was 14.2 ± 0.6 pmol/min per mm compared to LPS at 9.4 ± 0.4 pmol/min per mm, p < 0.01). These results demonstrate that similar to signaling in mTAL^8^, luminal exposure to LPS inhibits bicarbonate absorption in the OMCDi via a TLR4-PI3-kinase dependent pathway.

## Discussion

The key finding of this study is that either luminal or basolateral exposure of the isolated perfused OMCDi to LPS, a major *E. coli* cell wall constituent, reversibly inhibits bicarbonate absorption. Results presented herein are the first to show that LPS signaling targets acid–base transport mechanisms in the OMCDi. In the mTAL luminal LPS-induced signaling via TLR4 inhibits basolateral sodium/hydrogen exchange (NHE) activity^8,19^, whereas basolateral TLR4 signaling, which is TLR2 dependent^20^ targets apical NHE3 activity^19^. Consistent with the results of Good and colleagues^8^ luminal TLR4 signaling in the OMCDi inhibited bicarbonate absorption via a PI3-K dependent pathway; the PI3-K inhibitor wortmannin blocked much of the effect of luminal exposure to LPS (see Fig. 3). However the target of TLR4 signaling in the OMCDi is distinct, as bicarbonate absorption in this segment is mediated by the combined action of basolateral chloride/bicarbonate exchange (AE1) and an apical B1-V-ATPase. Consistent with the apical H^+^-ATPase activity in α-ICs being the target of TLR4-induced signaling in the OMCDi luminal exposure to LPS regularly reduced transepithelial voltage created by the electrogenic secretion of protons, and the change in voltage was mitigated in the presence of wortmannin (Table 1).

MPLA is a detoxified derivative of LPS as well as a partial TLR4 agonist that effectively blunts pathophysiological responses to LPS^21^. The systemic toxicity of MPLA compared to native LPS is estimated to be 99% reduced^22^. Because it can enhance the adaptive immune response with a minimum of inflammatory side effects, MPLA has been used as an immunoadjuvant in humans^12,22^. Pretreatment with MPLA induces resistance to endotoxemia in animals and humans^21,23–25^. Good and colleagues reported that MPLA induces TLR4 signaling via a TRIF-PI3K-AKT pathway that prevented LPS induced ERK activation^12^. Basolateral exposure of the OMCDi to MPLA and wortmannin likely blocked the effect of luminal LPS on bicarbonate absorption (Fig. 2) and transepithelial voltage via a similar mechanism (see Table 1). Thus, these agents may prevent OMCDi dysfunction associated with pyelonephritis. The medullary collecting duct is the first nephron segment exposed to *E. coli* pyelonephritis during an ascending UTI, and so this result provides proof-of-concept for attenuation of pyelonephritis via pharmacologic interventions that target basolateral TLR4 signaling. Indeed, partial TLR4 agonists represent a viable antibiotic-sparing therapy for treatment of acute pyelonephritis^26–29^.

An association between abnormalities in electrolyte and acid–base balance and acute pyelonephritis is common in young children^9^. Recent studies in our laboratory have focused on the intersection of metabolic acidosis and innate immune defense^30,31^. Intracellular acidification promotes the accumulation of 2-hydroxyglutarate, which in turn triggers HIF-1 stabilization via prolyl hydroxylase inhibition^32^. HIF-1 elicits adaptive responses to both acidosis and microbial invasion via induction of SDF-1 (CXCL12) and antimicrobial peptide expression, respectively^30,33,34^. Despite HIF-dependent upregulation of AMPs, metabolic acidosis markedly impairs clearance of urinary tract infection with uropathogenic *E. coli* (UPEC-UTI) and thus exacerbates pyelonephritis in innate immune competent C3H strains mice that are prone to vesicoureteral reflux (VUR)^35^. Although acidification of culture media or urine (pH ≤ 6) limits bacterial growth in vitro^36^, urine acidification per se was not a major contributor to clearance of UPEC-UTI in this study as neutralization of urine in the setting of metabolic acidosis via concurrent administration acetazolamide did not affect UPEC burden^35^. UPEC burden in TLR4-deficient mice (C3H-HeJ) mice was unaffected by acidosis suggesting that metabolic acidosis impairs some aspect of the TLR-4-dependent innate immune response. Collectively, these studies suggest that other aspects of pathophysiology associated with metabolic acidosis impair clearance of UPEC UTI and thus supersede any benefit of AMP production and urine acidification by α-ICs^36^.

Thus, acidosis represents a key comorbidity with *E. coli* pyelonephritis and this association may be explained, at least in part, by LPS-induced TLR4 signaling that inhibits bicarbonate absorption by the OMCDi. MPLA and PI3-K inhibition may mitigate acidosis as well as OMCDi injury induced by LPS. Finally, formal correction of acidosis may speed recovery from urinary tract infections and thus represent a key antibiotic-sparing therapy adjunct for treatment of acute pyelonephritis.
